# Highly Sensitive Photothermal Fiber Sensor Based on MXene Device and Vernier Effect

**DOI:** 10.3390/nano12050766

**Published:** 2022-02-24

**Authors:** Qing Wu, Si Chen, Lixin Guan, Haibin Wu

**Affiliations:** 1 Heilongjiang Province Key Laboratory of Laser Spectroscopy Technology and Application, Harbin University of Science and Technology, Harbin 150080, China; wuqing@hrbust.edu.cn; 2 School of Physics and Electronic Information, Gannan Normal University, Ganzhou 341000, China; chensics9@163.com (S.C.); lxguan@gnnu.edu.cn (L.G.)

**Keywords:** tapered fiber, two-dimensional material, fiber optic sensor, Vernier effect

## Abstract

A photothermal fiber sensor based on a microfiber knot resonator (*MKR*) and the Vernier effect is proposed and demonstrated. An *MXene* Ti_3_C_2_T_x_ nanosheet was deposited onto the ring of an *MKR* using an optical deposition method to prepare photothermal devices. An *MXene* *MKR* and a bare *MKR* were used as the sensing part and reference part, respectively, of a Vernier-cascade system. The optical and photothermal properties of the bare *MKR* and the *MXene* *MKR* were tested. Ti_3_C_2_T_x_ was applied to a photothermal fiber sensor for the first time. The experimental results showed that the modulation efficiency of the *MXene* *MKR* was 0.02 nm/mW, and based on the Vernier effect, the modulation efficiency of the cascade system was 0.15 nm/mW. The sensitivity was amplified 7.5 times. Our all-fiber photothermal sensor has many advantages such as low cost, small size, and good system compatibility. Our sensor has broad application prospects in many fields. The proposed stable *MKR* device based on two-dimensional-material modification provides a new solution for improving the sensitivity of optical fiber sensors.

## 1. Introduction

Fiber optic sensors have the advantages of a large dynamic range, high sensitivity, good system compatibility, and a compact structure, and they are an attractive technology that has a wide range of application prospects in the fields of detection, medical diagnosis, and environmental monitoring [1]. The basic structure of a fiber optic sensing system includes a light source, an incident fiber, a modulation region, an exit fiber, and a detector. A fiber optic sensing element modulates an optical signal (intensity, frequency, wavelength, phase, polarization, etc.) according to changes in the modulation zone. The structures commonly used in phase fiber sensors include interferometer and resonator structures. Optical fiber sensors with the interferometer structure often use a tapered-fiber-based sensing device. Tapered fiber has the advantages of low loss, a high evanescent field, and easy integration, and is a breakthrough in the fabrication of miniaturized photonic devices, including phase fiber sensors [2]. Tapered-fiber-based photonic device microfiber knot resonators (*MKRs*, tapered fiber knotting) are one of the research hotspots in the field of phase fiber sensors. *MKR*s have the advantages of strong anti-interference, a fast response speed, high resolution, a small size, and stable measurement; thus, they have attracted much attention in the field of fiber optic sensors [3,4].

Due to the development of science and technology, the sensitivity of sensors is increasing. As an effective method to improve sensitivity, the Vernier effect has been widely used to improve the accuracy and sensitivity of measuring instruments, such as barometers, temperature sensors, humidity sensors, refractive index sensors, and so on [5,6]. The Vernier effect in the field of fiber optic sensors is usually in the form of cascading and parallel interference structures. Fiber optic interferometers include the Mach–Zehnder interferometer (MZI), the Michelson interferometer (MI), the Fabry–Perot interferometer (FPI), and the Sagnac interferometer (SI). It is well-known that waveguides, highly nonlinear fibers, fiber gratings, and the in-line MZI have the disadvantages of high cost and poor compatibility and tunability when applied to sensors. MZIs have large losses and mode interference, which cause spectrum confusion. MZI and MI structures are extremely sensitive to disturbances from ambient temperature and stress, resulting in poor environmental stability [7]. *MKR*-based devices have high reliability, which facilitates stability of the system; low loss and high coupling efficiencies, which facilitate compatibility with fiber optic systems; and a small volume, which facilitates driving production and system integration. These devices are suitable for a Vernier-cascade system and have been widely discussed [8].

Two-dimensional, nanomaterial-modified *MKRs* provide new ideas for fiber optic sensors [9,10]. Two-dimensional materials have been gradually explored in academic research concerning their practical applications [11,12,13,14]. *MXenes*, a rapidly developing two-dimensional material family, have been reported in many studies since the first successful preparation of *MXene* Ti_3_C_2_T_x_ in 2011 [15]. In 2015, *MXene* was first applied in the field of optics, and in 2017, it was applied in the field of sensors [16]. Many experimental and theoretical studies revealed the great application potential of the *MXenes* family in the fields of optoelectronics, photonics, catalysis, and energy [17,18,19]. The photothermal conversion efficiency of *MXene* Ti_3_C_2_T_x_ is ~100%, and the thermal conductivity coefficient of *MXene* Ti_3_C_2_T_x_ is 56 WM^−1^K^−1^, which expands its application to photothermal-related fields such as thermal management and photothermal fiber sensors [20,21].

We present a highly sensitive photothermal fiber sensor based on an MXene-Ti_3_C_2_T_x_-deposited *MKR* and bare *MKR* cascade. *MXene* Ti_3_C_2_T_x_ was fabricated using an aqueous acid etching method and deposited onto the ring of an *MKR* utilizing an optical deposition method. The spectral response of the *MXene*-deposited *MKR* was investigated, showing good resonant properties and a good photothermal response. When a control light (a 980 nm pump that the laboratory can provide) was shone onto the ring of the *MXene* *MKR* through a collimator, the *MXene* Ti_3_C_2_T_x_ absorbed the light and generated heat. A large amount of heat changed the refractive index of the device, thereby changing the phase of the signal light. The *MXene* *MKR* was the sensing part of the cascade system and the bare *MKR* was the reference part. The photothermal modulation efficiency of the sensing part was 0.02 nm/mW and that of the cascade system was 0.15 nm/mW. The corresponding sensitivity was amplified 7.5 times. Our proposed integrated device, *MXene-MKR*, is small in size and suitable for packaging in compact single-unit systems, highlighting its potential for providing a new method for high-sensitivity optical sensing applications.

## 2. Material Characterization and Device Fabrication

### 2.1. Material Characterization

MXene Ti_3_C_2_T_x_ was prepared via an aqueous acid etching method [22,23]. At room temperature, 40 mL of 40 wt % HF aqueous solution was mixed with Ti_3_AlC_2_ MAX powder (1 g) and stirred for 20 h. After using corrosion technology, we rinsed the precipitate in the mixed solution with deionized water to ensure that the pH value of the solution was greater than 6. Then, the Ti_3_C_2_T_x_ solution was centrifuged at 18,000 rpm for 30 min. The supernatant was removed to retain the precipitate, and the precipitate was dried in a vacuum drying oven for later use. A simple and gentle exfoliation named the freeze-and-thaw method was used to prepare Ti_3_C_2_T_x_ nanosheets [24]. We added 40 mg as-prepared *MXene* power to 40 mL deionized water and stirred the dispersion for 10 min. Afterwards, the dispersion was placed in a refrigerator at 4 °C for cooling; then, it was placed in a refrigerator at −20 °C for several hours. We took out the frozen dispersion and thawed it naturally at room temperature. Then, the *MXene* suspension was treated with an ultrasonic water bath for 1 h. This freeze-and-thaw process was repeated several times to improve the yield of Ti_3_C_2_T_x_ nanosheets. Finally, the suspension was centrifuged at 3000 rpm for 30 min; then, it was centrifuged at a speed of 18,000 rpm for 30 min. Final nanosheets from the Ti_3_C_2_T_x_ dispersion were obtained by eliminating the additional solution and redispersing the sediment in water.

**Figure 1:**
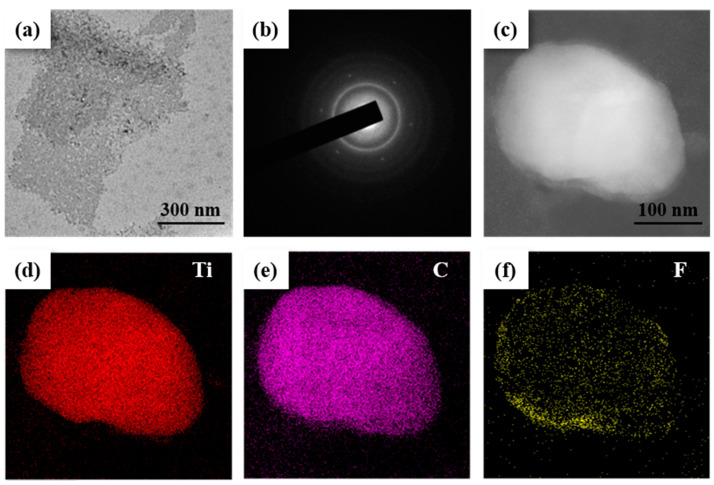
Characterization of *MXene* Ti_3_C_2_T_x_: (**a**) TEM image of the Ti_3_C_2_T_x_ nanoflakes; (**b**) SAED patterns of the Ti_3_C_2_T_x_ nanoflakes; (**c**) STEM of a Ti_3_C_2_T_x_ nanoflake; (**d**–**f**) elemental maps of the nanoflake in Figure (**c**), which were obtained from the EDS results.

Characterization methods were used to study the MAX phase material, and it was successfully exfoliated into *MXene* nanosheets. Transmission electron microscopy (TEM), selected area electron diffraction (SAED), and energy dispersive spectrometer (EDS) images were employed to investigate the characterization of the delaminated Ti_3_C_2_T_x_ nanosheets. The morphology of the delaminated nanosheets was studied by TEM. The morphology of the final product was investigated using TEM, and the result is presented in Figure 1a, showing that it was a nanosheet structure with lateral dimensions of 450–714 nm. Moreover, the crystal structure of the nanosheets was further studied by SAED. The results are shown in Figure 1b, indicating that the hexagonal-structure nanosheets had high crystallinity [25,26,27]. EDS was used to investigate the elemental distribution of *MXene* nanosheets. STEM was used to record the morphology of an investigated nanosheet; then, EDS was used to measure the elemental distribution of the nanosheet. The elemental mapping (Figure 1d–f) shows that Ti, C, and F elements are evenly distributed throughout the nanosheet range in Figure 1c, indicating that the Ti_3_C_2_T_x_ nanosheets were successfully prepared. The F element belonged to the termination group of *MXene* and was caused by the HF etching process.

**Figure 2:**
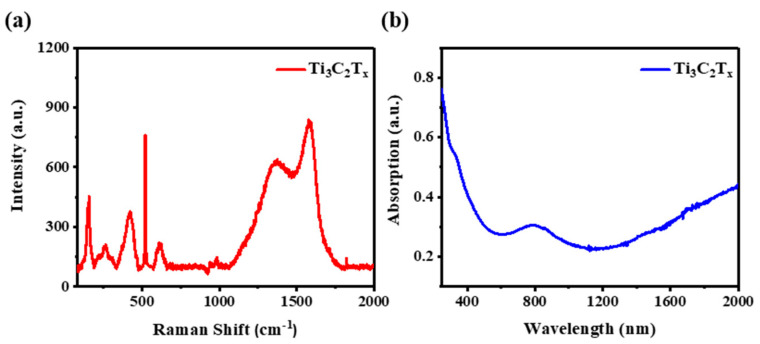
(**a**) the Raman spectrum of Ti_3_C_2_T_x_; (**b**) UV–Vis–NIR absorption spectrum.

A Raman spectrum (Figure 2a) of Ti_3_C_2_T_x_ was used to further study the structure of the nanosheets. For Ti_3_C_2_T_x_, characteristic peaks at 154.8, 264.3, 425, and 609.3 cm^−1^ have been previously observed [28,29]. Regarding our nanosheets, a characteristic peak at 157 cm^−1^ belonged to TiO_2_, which was possibly caused by oxidation of the Ti_3_C_2_T_x_ surface during the preparation process. The Raman spectrum further illustrated that *MXene* nanosheets were successfully fabricated. Furthermore, the linear absorption property of Ti_3_C_2_T_x_ was investigated, and the result is presented in Figure 2b. The absorption spectrum demonstrates the ultrabroad absorption properties of the nanosheets ranging from 250 to 2000 nm, which indicates that this *MXene* material can operate under a wide range of wavelengths. This was attributed to the narrow band gap of Ti_3_C_2_T_x_ [30]. 

### 2.2. Devices

**Figure 3:**
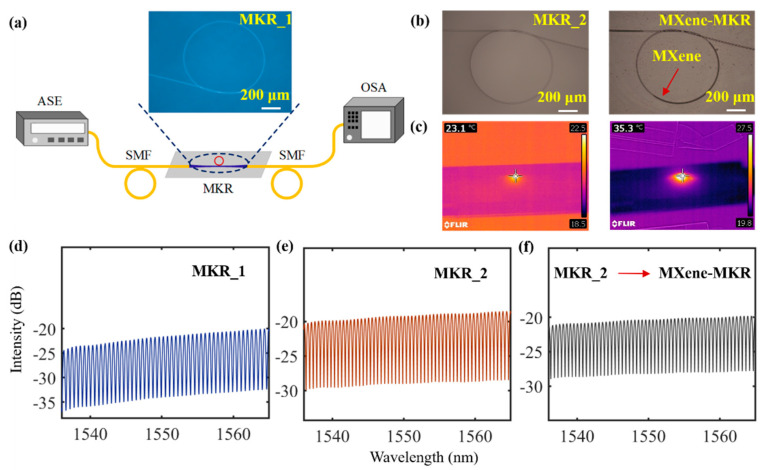
(**a**) Diagram of system apparatus for testing spectral response; (**b**) optical microscope image; (**c**) thermograms of bare *MKR* and *MXene* *MKR*; transmission spectra of (**d**) *MKR*_1, (**e**) *MKR*_2, and the (**f**) *MXene* *MKR*.

*MXene*-deposited tapered fiber is an important component in the proposed photothermal fiber sensor. Tapered fiber with waist length of 1–2 cm and waist diameter of 1–3 μm was prepared using the oxyhydrogen flame method. Tapered fiber has a long and thin waist with a good toughness that is conducive to knotting. *MKR* was prepared by knotting the tapered fiber. Two *MKR*s were prepared, and their spectral responses were tested using a broadband amplified spontaneous emission (ASE OS8143 China) source as shown in Figure 3a. This illustration corresponds to a microscope picture of the *MKRs*, where *MKR*_1 and *MKR*_2 had diameters of 900 and 1050 μm, respectively, and the corresponding tapered fiber diameter was 3μm. The *MXene-MKR* was prepared by coupling between the near-field surface of tapered fiber and *MXene* Ti_3_C_2_T_x_. An optical deposition system is shown in Figure 3a. The output power of the ASE source was set at 40 mW, and a *MXene* dispersion liquid dropped to the vicinity of the *MKR* and slowly spread to the *MKR* ring. Due to the strong evanescent field of the tapered fiber, the material was adsorbed to the surface of the tapered fiber. In the process of material deposition, the deposition length of material and the loss of device were recorded in real time, and the deposition parameters were controlled by the power of the light source and the amount of material. Optical microscope images of the *MXene* before and after deposition are shown in Figure 3b. The photothermal effect of the *MXene-MKR* was measured utilizing infrared thermal imaging (FLIR E60 USA ) of the control light (980 nm pump light). The control light was irradiated onto the ring of the *MKR* using infrared thermal imaging (FLIR E60), which measured the photothermal effect of the device. As shown in Figure 3c, the temperatures of the bare *MKR* and *MXene-MKR* were 23.1 and 35.3 °C, respectively, at a pump power of 200 mW. This shows that *MXene* played a key role in raising the temperature. The transmission spectra of the *MKR*s are shown in Figure 3d–f. The free spectral ranges (*FSRs*) corresponding to *MKR*_1 and *MKR*_2 were 0.585 and 0.505 nm, respectively, according to the formula *FSR* = λ^2^/(π*D*·*n_ef__f_*), where λ is an incident wavelength, *D* is the diameter of the *MKR*, and *n_eff_* is an effective refractive index [31]. The calculated *FSRs* of *MKR*_1 and *MKR*_2 were 0.586 and 0.502 nm, respectively, which is consistent with our experimental results. As *MKR*_2 had a larger diameter and was more conducive to deposition materials, we chose *MKR*_2 as the sensing device and *MKR*_1 as the reference device. Figure 3f is a transmission spectrum of *MXene*-deposited *MKR*_2. The transmission spectrum shows that the loss of the device increased and the resonant wavelength shifted before and after deposition.

## 3. Experimental Setup and Results

### 3.1. Experimental Setup

**Figure 4:**
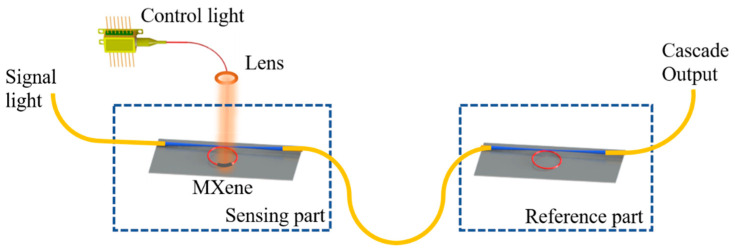
Experimental setup of photothermal fiber sensor based on *MKR* and *MXene-MKR* cascade.

The configuration of our photothermal fiber sensor is shown in Figure 4, where *MKR*_1 is the reference part, the *MXene-MKR* is the sensing part, and the *FSRs* of the *MKRs* are *FSR_MKR_* and *FSR_MXene-MKR_*. The signal light was 1550 nm, generated by an ASE light source, and the control light was 980 nm, generated by a pump light source (can be provided). The output of the system was connected to an optical spectrum analyzer (OSA, Yokogawa AQ6370D, Japan). The control light irradiated *MXene* Ti_3_C_2_T_x_ onto the ring of the *MXene-MKR* through a collimator, and the *MXene* Ti_3_C_2_T_x_ absorbed the pump light. The photothermal effect of *MXene* Ti_3_C_2_T_x_ is a large amount of heat production, and the thermo-optical effect is a change in the refractive index of the sensor, thereby changing the phase of the signal light. The corresponding transmission spectrum is moved. The introduction of the optical Vernier effect can greatly improve sensor sensitivity, and the sensor sensitization effect can be realized by amplifying the *FSR* value of the sensing part. Vernier calipers are widely used in high-precision and short-distance ranging. The Vernier effect refers to a large change in the range of alignment indexing caused by small measurement changes. There are some similar physical parameters in the form of scale in optics. For example, the transmission spectrum of a broad-spectrum light source through an *MKR* is a comb spectrum. The peak spacing of the two *MKR*s produces a minimal difference, and the two devices can be cascaded to create an optical Vernier effect. A small shift in the spectrum of one device magnifies the spectral shift of the cascade system several times, which improves measurement sensitivity while maintaining a larger measurement range. When the resonator *MXene-MKR* structure is modulated by an external sensor signal, the *MXene* *MKR* and *MKR*_1 cascade and the Vernier interference fringes drift. The amount of stripe movement is far more than the drift of the spectral fringes of the *MXene-MKR*, which is the basis for realizing high-modulation-efficiency sensing.

Based on the Vernier effect [32], the *FSR* of the envelope can be defined as:

(1) $${\mathrm{FSR}}_{\mathrm{envelope}}=\frac{{\mathrm{FSR}}_{\mathrm{MKR}}\cdot {\mathrm{FSR}}_{\mathrm{MXene}-\mathrm{MKR}}}{\left|{\mathrm{FSR}}_{\mathrm{MKR}}{-\mathrm{FSR}}_{\mathrm{MXene}-\mathrm{MKR}}\right|}=\frac{\lambda^{2}}{\pi \cdot n_{eff}\left|D_{\mathrm{MKR}}-D_{\mathrm{MXene}-\mathrm{MKR}}\right|}$$

Compared to the phase shift of *MKR*_1, the phase shift of the cascade sensor is amplified by *K_s_* times, and the value of *K_s_* is:

(2) $$K_{s}=\frac{{\mathrm{FSR}}_{\mathrm{MKR}}}{\left|{\mathrm{FSR}}_{\mathrm{MKR}}{-\mathrm{FSR}}_{\mathrm{MXene}-\mathrm{MKR}}\right|}=\frac{D_{\mathrm{MXene}-\mathrm{MKR}}}{\left|D_{\mathrm{MKR}}-D_{\mathrm{MXene}-\mathrm{MKR}}\right|}$$

The spectrum of the cascaded sensor is composed of a fine-comb spectrum and a large envelope. Firstly, at least one complete envelope period must be observed within the limited wavelength range of the light source. Secondly, the cascaded superposition spectrum must be regular and periodic, that is, both *FSR_MKR_* and *FSR_MXene-MKR_* must be regular and periodic. A cascade system based on the Vernier effect needs to satisfy the condition that the sensor is sensitive to variables while the reference part is insensitive to variables. According to the above analysis, the closer the *D_MKR_* and *D_MXene-MKR_* values, the higher the sensing modulation efficiency.

### 3.2. Experimental Results

**Figure 5:**
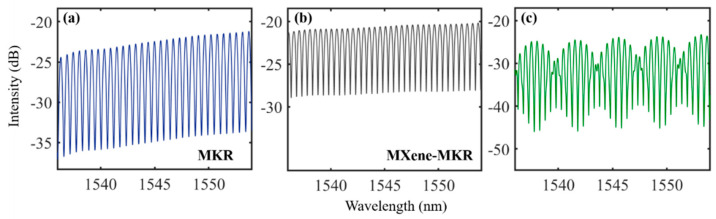
Transmission spectra of the (**a**) *MKR*; (**b**) *MXene-MKR*; (**c**) cascade system.

**Figure 6:**
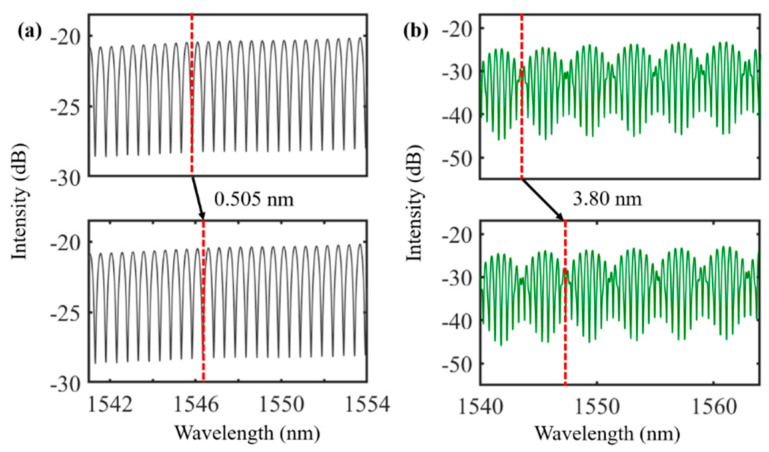
Wavelength shift of (**a**) *MXene-MKR* and (**b**) the cascade system at a pump power of 34 mW.

In the cascade system, the transmission spectra of the reference part and the sensing part are shown in Figure 5a,b, respectively, and the corresponding *FSRs* were 0.585 and 0.505 nm, respectively. The transmission spectrum of the cascade system is shown in Figure 5c, and the *FSR* was 3.73 nm. A pump light generated by a laser diode was directly illuminated by a cylindrical lens pointed toward the ring of the *MKR* with *MXene* Ti_3_C_2_T_x_. Due to a photothermal effect, the material absorbed the 980 nm pump light, producing a large amount of heat and changing the refractive index of the device. The transmission spectrum of *MXene-MKR* achieved a phase shift when a pump light was applied to a material-based device. When the pump power was 34 mW, the transmission spectrum moved 0.505 nm in the long wavelength direction, and the corresponding cascading system moved 3.8 nm (as shown in Figure 6).

**Figure 7:**
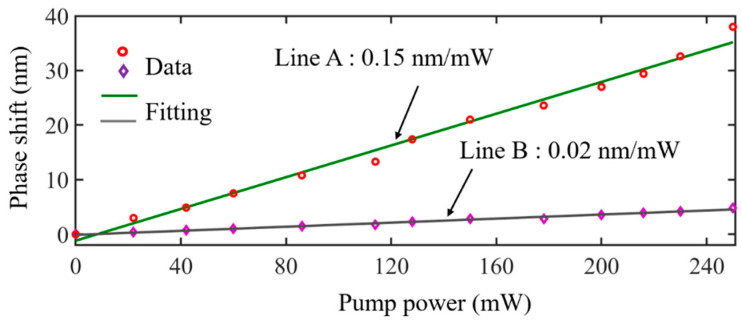
Modulation efficiency of (Line A) *MXene-MKR* and (Line B) cascade system.

When the pump power was increased from 0 to 250 mW (which was the maximum power), *MXene-MKR*’s transmission spectrum (without the Vernier effect) moved 4.8 nm, and the corresponding modulation efficiency was 0.02 nm/mW. For a cascading system of the entire Vernier, when the optical intensity was increased from 0 to 250 mW, the long wavelength direction moved 38 nm, the modulation efficiency of the cascade system was 0.15 nm/mW (as shown in Figure 7), and the calculated *K* of the system was 7.5, which is in line with the theoretical value (*K*_s_ = 7.49).

## 4. Discussion

In this work, an all-fiber cascade *MKR* and *MKR-MXene* photothermal sensor were constructed. This sensor has advantages including small size, low power consumption, and good compatibility, and it is economical as well. This study is the first time that *MXene* Ti_3_C_2_T_x_ with a high photothermal conversion efficiency has been applied to the field of photothermal sensors. Our selection of photothermal materials takes into account the advantages of *MXene* Ti_3_C_2_T_x_, such as high thermal conductivity, a high photoinduced damage threshold, greater flexibility, good electrical conductivity, good tunability of the electronic band structure, good broadband saturation absorption, and so on, and is conducive to the preparation of photothermal devices. The photothermal conversion efficiency of *MXene* Ti_3_C_2_T_x_ is ~100%, which is beyond the reach of other two-dimensional materials. In the context of structure and devices, the damage threshold of *MXene* Ti_3_C_2_T_x_ loaded on tapered fiber is high, and the device is stable. Both the structure coupling and interference mechanism of *MKR*s are wavelength-sensitive, which is beneficial to their application in the sensing field.

Compared to MZI- and MI-type tapered fiber optic sensors, the *MKR* structure adopted by our system has better stability performance. It also has a simpler structure and is easier to make as an integrated sensor. The MZI and the MI have the problems of wavelength drift and environmental instability, and such systems have too many devices, which leads to a large footprint and is not conducive to integration. For the sensitivity of our system, the diameter of the *MKR* can be adjusted to obtain greater sensitivity. The taper fiber prepared for the *MKR* has a smaller diameter, and the sensitivity of *MXene* Ti_3_C_2_T_x_ per unit length is greater than that of photothermal fiber sensors with interferometer structures.

Due to the limitations of experimental conditions, only a 980 nm pump light was used to control the light. The absorption spectrum of *MXene* Ti_3_C_2_T_x_ is 250–2000 nm, which can realize photothermal sensing in different bands. The family of *MXenes* aids the development and exploration of new materials and allows us to obtain required materials through functional group regulation, providing a new idea for optical sensors based on two-dimensional materials. As *MKR*s can be used in formats such as parallel, series, and other ways, later works may prepare a multipoint, high-sensitivity, multiparameter sensor system. As a functional, two-dimensional material, *MXenes* provide an economical and practical platform for creating high-efficiency fiber sensors with antielectromagnetic interference and good compatibility.

## 5. Conclusions

In summary, *MXene* Ti_3_C_2_T_x_ was successfully prepared using a HF acid etching method. The wide spectrum absorption of *MXene* Ti_3_C_2_T_x_ shows its potential to be used in the fabrication of photonic devices. We demonstrated a sensitivity-enhanced cascade photothermal fiber sensor based on *MXene* Ti_3_C_2_T_x_. *MKR-MXene* was the sensor device, the control light was a 980 nm pump light, and the corresponding modulation efficiency was 0.02 nm/mW. A bare *MKR* was used as the reference part. After the system cascaded, the modulation efficiency was 0.15 nm/mW, and the sensitivity was increased 7.5 times, which is consistent with the theoretical value. The preparation process of *MXene* Ti_3_C_2_T_x_ is relatively mature, and this material has a high photothermal conversion efficiency, a wide spectrum detection range, and a large population. *MXene* Ti_3_C_2_T_x_ has great application potential in optoelectronic devices. Our proposed device based on an *MKR* using *MXene* Ti_3_C_2_T_x_ has a small footprint, is suitable for packaging and integration, and highlights a new idea for high-performance optical sensing applications.

## Data Availability

Not applicable.
